# Carbon Monoxide Poisoning Presenting With Transient Visual Field Defects

**DOI:** 10.7759/cureus.106180

**Published:** 2026-03-31

**Authors:** Satya Naga Shravan Chilukuri, Harish Krishnappa

**Affiliations:** 1 Internal Medicine, Morriston Hospital, Swansea, GBR

**Keywords:** bitemporal hemianopia, carbon monoxide intoxication, chiasmal hypoxia, heavy smoking, microvascular ischemia, optic chiasma

## Abstract

Carbon monoxide (CO) toxicity typically presents with drowsiness, headache, and neurological disturbances. Visual field defects involving the optic chiasma are very rarely seen.

A case of a 31-year-old female, with a history of heavy smoking and learning disability, presented to the acute medical ward with 3-4 days of frontal headache, neck pain, room spinning sensation, and an episode of loss of consciousness. Neurological examination revealed a bitemporal hemianopia that progressed to a temporal hemianopia over the next day and completely resolved within 48 hours.

Blood gas analysis showed a high carboxyhaemoglobin (COHb) level of 9.7%, with normal pH and lactate. The CT brain showed no acute abnormality. Ophthalmology confirmed visual field defects with no diplopia. Neurology advised an MRI to rule out pituitary apoplexy, and the image showed no structural abnormality.

The transient visual field changes were consistent with possible CO toxicity-induced hypoxia affecting the optic chiasma or its vascular supply. Symptoms quickly resolved after oxygen therapy and abstinence from smoking.

This study highlights the diagnostic challenge of identifying CO toxicity in heavy smokers and their atypical clinical manifestations. It emphasises the need to consider toxic exposure when presenting with fluctuating visual field defects and particularly, when their neuro-imagining is normal. Environmental sources of CO exposure were assessed, and no clear source was identified, with significant tobacco use considered a potential contributing factor.

## Introduction

Carbon monoxide (CO) is a colourless, odourless gas produced by the incomplete combustion of carbonaceous material [1]. It binds to the iron moiety of haemoglobin with approximately 240 times the affinity of oxygen, thereby reducing oxygen affinity and the disruption of mitochondrial respiration [2].

Clinical presentation in patients with CO poisoning ranges from headache and dizziness to coma and death [3]. Neurological sequelae are well recognised; however, visual disturbances are less commonly reported and are typically attributed to cortical injury, optic neuropathy, or delayed neurological sequelae. Previously described neuro-ophthalmic manifestations include optic neuropathy and cortical visual impairment, whereas involvement of the optic chiasm producing bitemporal visual field defects is extremely rare, with only isolated cases reported in the literature [4]. Bitemporal hemianopia usually raises concern for pituitary apoplexy, a mass lesion, or suprasellar pathology [5]. However, transient visual field defects secondary to hypoxia have been discovered in isolated cases of CO toxicity [6].

CO poisoning is a common cause of accidental poisoning and can result in significant neurological morbidity [3].

Carboxyhaemoglobin (COHb) levels are typically less than 2% in non-smokers but may reach up to 5-10% in heavy smokers, which can complicate interpretation in symptomatic patients.

We present an unusual case of reversible bi-temporal hemianopia in a heavy smoker with suspected CO toxicity, illustrating a unique neuro-ophthalmic manifestation and a potential diagnostic pitfall.

## Case presentation

A 31-year-old female with a learning disability presented with a 4-day history of frontal headache, neck pain, vertigo and an episode of loss of consciousness for a few minutes. She lived alone in a bungalow and smoked approximately 2 grams of tobacco a day. The patient was questioned regarding possible environmental sources of CO exposure, including household heating systems, gas appliances, and enclosed-space exposure. No clear environmental source was identified. The patient reported significant tobacco consumption, which may have contributed to the elevated COHb level.

On examination, her vitals were stable. She was alert and oriented. Blood investigations were normal, except that venous blood gas analysis revealed an elevated COHb level (Table 1).

**Table 1:** Venous blood gas analysis Venous blood gas analysis performed on admission demonstrating an elevated carboxyhaemoglobin (COHb) level in the setting of otherwise normal acid–base parameters and lactate. Reference ranges for each parameter are provided for comparison.

Parameter	Result	Reference Range (Adult)	Interpretation
pH	7.39	7.35-7.45	Normal
pCO₂	5.3	4.6-6.0 kPa (venous)	Normal
pO₂	4.5	4.0-6.0 kPa (venous)	Within the expected venous range
HCO₃⁻	23	22-26 mmol/L	Normal
O₂ saturation	68.5	60-80% (venous)	Within the expected venous range
Oxyhaemoglobin	61.6	60-80% (venous)	Within the expected venous range
Carboxyhaemoglobin	9.7	<2% (non-smoker); <5-10% (smoker)	Elevated
Lactate	0.7	0.5-2.2 mmol/L	Normal

Cardiovascular, respiratory, gastrointestinal and musculoskeletal examinations were normal. Neurological examination revealed a bitemporal hemianopia, with preserved motor and sensory function.

On the following day, it evolved to left temporal hemianopia and resolved completely within 48 hours of admission.

**Figure 1:**
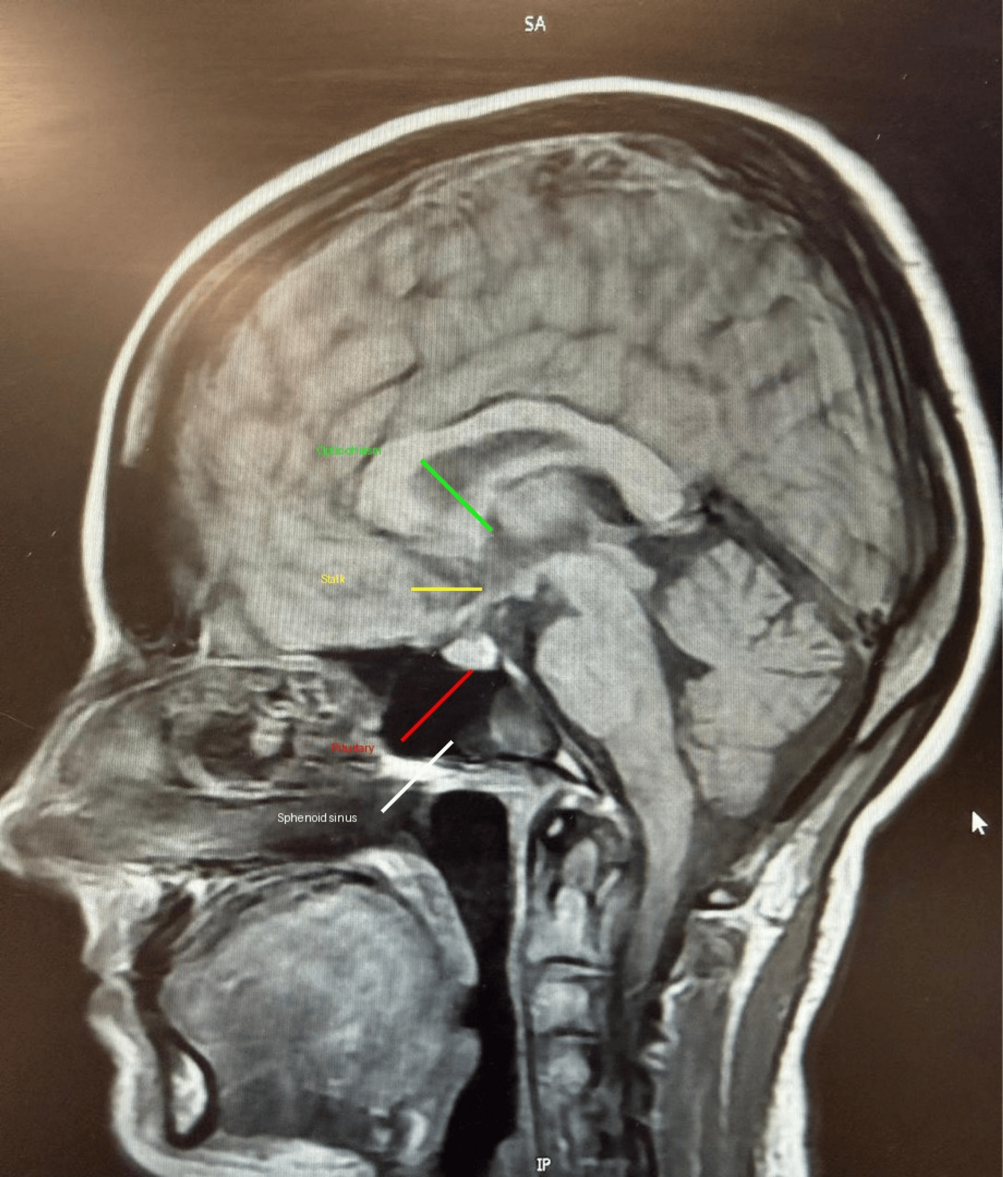
Sagittal MRI brain (midline view) Sagittal T1-weighted magnetic resonance imaging (MRI) of the brain demonstrates preserved midline anatomy. The pituitary gland is normal in size and contour, with no evidence of haemorrhage or mass lesion. There is no suprasellar extension or compression of the optic chiasm.

**Figure 2:**
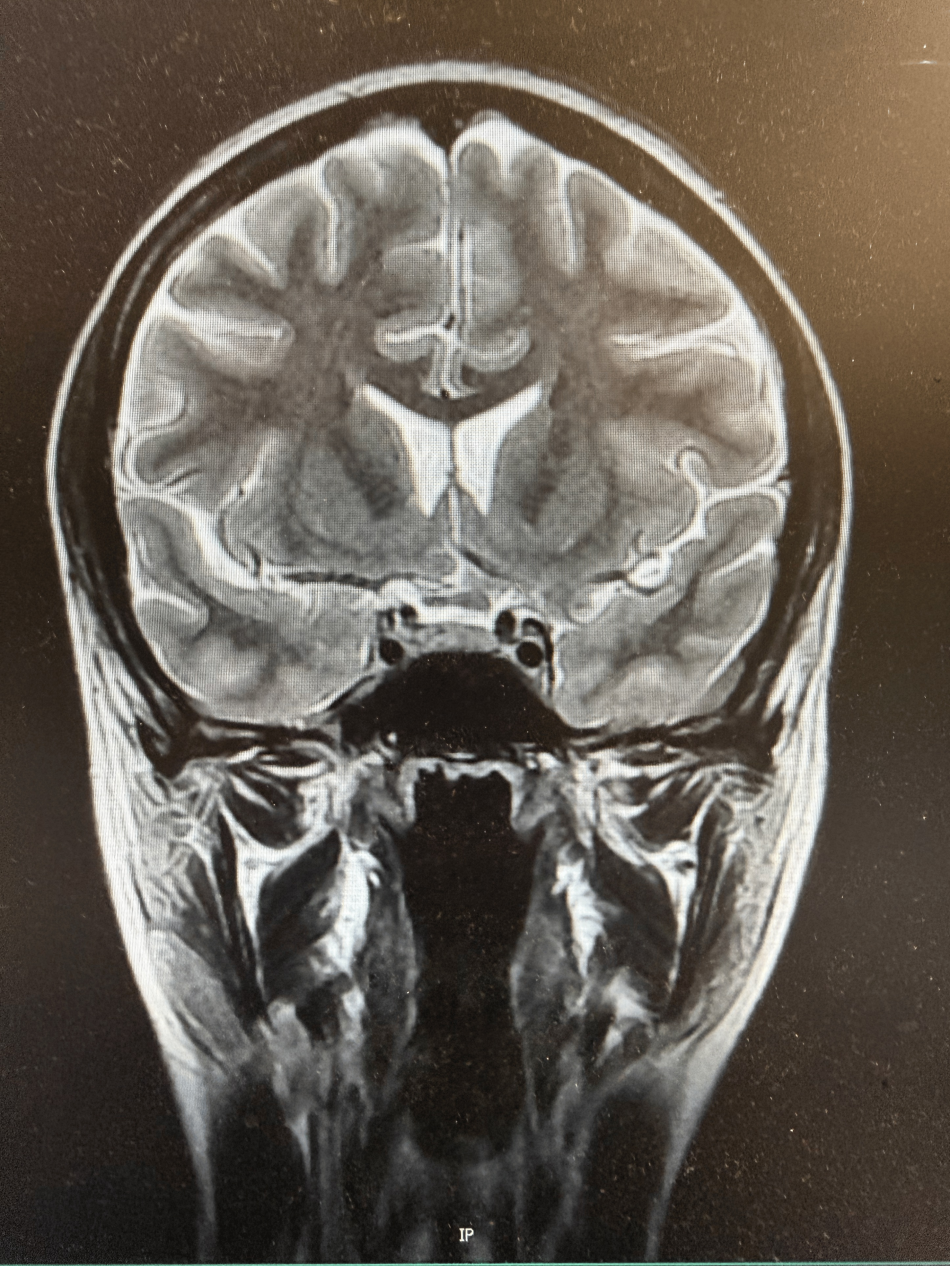
Coronal MRI through the sellar region Coronal T2-weighted MRI through the sellar and suprasellar region demonstrating normal pituitary morphology and intact optic chiasm. No sellar or suprasellar mass, haemorrhage or structural abnormality is identified.

The CT brain showed no acute abnormality. Visual field assessment was performed clinically by ophthalmology; however, formal automated perimetry was not available for inclusion in this report. Neurology raised the probability of pituitary apoplexy due to the pattern of visual disturbance. MRI brain demonstrated no evidence of sellar or suprasellar pathology (Figures 1, 2).

This patient was diagnosed with CO poisoning resulting in transient chiasmal dysfunction. High-flow oxygen therapy was initiated promptly following clinical suspicion of CO exposure. The patient’s visual symptoms began to improve within 24 hours and resolved completely within 48 hours of admission. She was discharged with community follow-up.

## Discussion

CO exerts its toxic effects primarily through tissue hypoxia and cellular respiratory dysfunction, leading to a spectrum of systemic and neurological complications [1,2]. Acute CO poisoning most commonly manifests with central nervous system symptoms, including headache, dizziness, weakness, seizures, and altered consciousness [2]. Visual disturbances are less frequently reported and are typically attributed to cortical involvement rather than anterior visual pathway dysfunction. This case highlights an unusual presentation of CO toxicity with transient neuro-ophthalmic deficits suggestive of chiasmal involvement.

Several pathophysiological mechanisms may explain the reversible bitemporal hemianopia observed in this patient. The optic chiasm is a metabolically active structure with high oxygen demand, rendering it particularly susceptible to hypoxic injury. CO binds haemoglobin with high affinity, impairing oxygen delivery and disrupting oxidative phosphorylation, which may result in transient neuronal dysfunction without structural damage [3,4]. In addition to systemic hypoxia, CO exposure is associated with endothelial injury and impaired cerebral autoregulation. Microvascular compromise and altered vasodilation may transiently reduce perfusion to the optic chiasm, further contributing to reversible dysfunction [3]. Retinal and optic nerve hypoxia may also play a contributory role, as retinal ganglion cells are highly sensitive to oxygen deprivation and may manifest as transient visual field defects [4,5].

Interpretation of COHb levels should be made in the context of clinical presentation. Although levels up to 5-10% may be observed in heavy smokers, symptomatic CO toxicity has been reported at similar concentrations, particularly in cases of sustained exposure or increased individual susceptibility to hypoxia. Additionally, measured COHb levels may underestimate peak exposure if sampling is delayed or if oxygen therapy has already been initiated.

In this patient, a COHb level of 9.7%, in conjunction with headache, vertigo, transient loss of consciousness, and fluctuating visual field deficits, was consistent with significant CO-related hypoxic stress [2]. Although this level may fall within the upper range reported in smokers, the presence of neurological symptoms suggests that even modest elevations can precipitate clinically meaningful cerebral hypoxia. Importantly, in the absence of identifiable environmental exposure, tobacco smoking alone can generate sufficient CO levels to impair cerebral oxygenation and provoke transient neurological manifestations [6]. The complete resolution of symptoms with high-flow oxygen therapy and the absence of structural abnormalities on neuroimaging is further suggestive of reversible hypoxic chiasmal dysfunction secondary to CO toxicity.

## Conclusions

This case highlights that carbon monoxide exposure may present with transient visual field defects mimicking chiasmal or pituitary pathology. Clinicians should consider carbon monoxide toxicity in patients presenting with fluctuating neuro-ophthalmic symptoms and normal neuroimaging. However, given that this is a single case report and that the observed carboxyhaemoglobin level may overlap with levels seen in smokers, a definitive causal relationship cannot be established, and the proposed mechanism remains speculative.
